# Supplementary material for: Why people select the outpatient clinic of medical centers: a nationwide analysis in Taiwan
Source: PeerJ. 2020 Aug 27;8:e9829. doi: 10.7717/peerj.9829 (PMC7456533; doi:10.7717/peerj.9829)
Supplement: Supplemental Information 3 [file peerj-08-9829-s003.pdf]

影響社群網站使用者對門診就醫選擇決定因素之調查

各位先生、女士您好：

政府推動分級轉診制度已多年，但各地區的大醫院門診仍然門庭若市，許多民眾對社區基層醫療信心不足。此問卷的主要目的在於瞭解「影響社群網站使用者對門診就醫選擇決定因素之調查」，作為協助政府規劃增強基層門診服務模式的參考，以助於分級醫療與轉診制度之落實，提升民眾門診看診品質。

本網路匿名問卷調查，問卷填寫過程則約需 5 至 10 分鐘，請您慎重考慮後自由決定是否參加，如您無意願參與此調查，請您直接點選回上一頁，即可離開本網頁。您的寶貴意見將使得本研究得以順利完成，在此感謝您的協助。

臺北榮民總醫院家庭醫學部 林明慧醫師敬上

第一部份　　門診就醫經驗

請依您最近兩年的門診就醫經驗圈選	非常好 5	4	3	2	非常不好 1	沒有經驗 0
1. 至基層診所門診的就醫經驗	5	4	3	2	1	0
2. 至一般醫院門診的就醫經驗	5	4	3	2	1	0
3. 至醫學中心門診的就醫經驗	5	4	3	2	1	0

4.請問你有信任的基層診所家庭醫師嗎? □1.有　□2.沒有

5.請問當你生病不知道該看哪一科時，你會優先選擇：

□1)基層診所門診　□2) 一般醫院門診　□3)醫學中心門診

6.請問當您有急性不適症狀時，你會優先選擇：

□1)基層診所門診　□2)一般醫院門診　□3)醫學中心門診

7.自 106/4/15 日起健保署調高未經轉診越級至醫院門診的部分負擔，請問對您的影響為：

□1)有影響，會改變門診就醫的習慣　□2)沒有影響，會維持原來的門診就醫習慣

8.你贊成小病至小診所看診，大病才至大醫院看診嗎?

□ 1)贊成　,原因為……(可複選)

- □A. 合理就診可以有效節省醫療資源的浪費
- □B. 避免輕症上大醫院排擠重症病人的照顧
- □C. 生病本來就該從最熟悉自己的家庭醫生看起
- □D. 大醫院醫師時間應該花在照顧住院病人

□ 2)不贊成,原因為……(可複選)

- □A. 一般病人無能力判斷病情輕重大小
- □B. 怕基層診所醫師經驗不足擔誤病情
- □C. 所在社區缺乏令人信賴的診所
- □D. 平常都習慣在大醫院看診拿藥
- □E. 基層診所開藥日數太短

第二部份　　門診就醫場所的選擇考量

I.請問當您有不適症狀時，選擇門診就醫場所的考量為？	非常重要 5	4	3	2	非常不重要 1
1. 考慮疾病的嚴重程度	5	4	3	2	1
2. 考慮醫療院所(診所或醫院)的知名度	5	4	3	2	1
3. 考慮醫療院所於媒體或社群網站之形象口碑	5	4	3	2	1
4. 醫療院所是否地點便利交通方便	5	4	3	2	1
5. 醫療院所是否有先進的檢查驗設備	5	4	3	2	1
6. 該醫療院所的藥物品質是否值得信任	5	4	3	2	1
7. 該醫療院所的專科科別是否多樣齊全	5	4	3	2	1
8. 該醫療院所的部分負擔費用是否較低	5	4	3	2	1
9. 該醫療院所醫師是否願意開立慢性病處方簽	5	4	3	2	1
10. 醫療院所其他醫療人員態度是否親切	5	4	3	2	1
11. 詢問親友是否推薦	5	4	3	2	1
12. 過去的就醫經驗	5	4	3	2	1
13. 等候時間不會太長	5	4	3	2	1
14. 醫師醫術是否高明	5	4	3	2	1
15. 醫師態度是否親切	5	4	3	2	1
16. 醫師看診是否不匆促	5	4	3	2	1
17. 醫師是否詳細解說回答	5	4	3	2	1
18. 醫師是否令人信任	5	4	3	2	1
19. 醫師的知名度	5	4	3	2	1
20. 醫師於媒體或社群網站之形象口碑	5	4	3	2	1

第三部份基本資料

1. 請問您本人是否罹患慢性病需定期至門診取藥：□1)是　　□2)否
2. 請問與你同住或熟識的家人，是否罹患慢性病需定期至門診取藥：□1)是　□2)否
3. 請問您的居住地：

1）台北市	3）桃園市	5）台南市
2）新北市	4）台中市	6）高雄市
7）六都之外的台灣北區	9）六都之外的台灣南區	11）花東離島其他地區
8）六都之外的台灣中區	10）宜蘭地區	

4.請問您的出生年：民國 _______年

5.請問您的教育程度：

□1)小學　□2)國中、初中　□3)高中、高職　□4)專科、大學　□5)研究所以上

6.請填寫您的性別：□ 1)男　□ 2)女

7.請問您目前的婚姻狀況是：□ 1)未婚　□ 2)已婚　□ 3)其他

8.請問您目前的職業是：

1）醫療相關
2）軍公教(不含醫療)
3）商業
4）工農漁業
5）服務業
6）自由業
7）家管
8）學生
9）已退休
10）其他

9.請問您個人的平均每月收入大約是：

□ 1）15,000 以下　□ 2）15,001~30,000　□ 3）30,001~50,000
□ 4）50,001~70,000　□ 5）70,001~90,000　□ 6）90,001 以上

問卷到此結束，非常感謝您的耐心回答！如果您有關於臺灣分級醫療與轉診制度的想法，歡迎提供您寶貴的建議：

