# Supplementary material for: Why people select the outpatient clinic of medical centers: a nationwide analysis in Taiwan
Source: PeerJ. 2020 Aug 27;8:e9829. doi: 10.7717/peerj.9829 (PMC7456533; doi:10.7717/peerj.9829)
Supplement: Supplemental Information 4 [file peerj-08-9829-s004.pdf]

The survey of factors associated with community websites users'decision on outpatient clinics for medical treatment

Good day, ladies and gentlemen.

Although the government has been promoting a hierarchy of medical care facilities consisting of four tiers: medical centers, regional hospitals, local community hospitals, and primary clinics. However, referral systems have not yet been successfully implemented. The outpatient clinics of medical centers are still packed with patients, and many people are not satisfied with primary medical care in their communities.

The main purpose of this questionnaire is to understand the " Why do people in Taiwan select the outpatient clinic of the medical center?". The result of this survey would be helpful for the implementation of the referral system and to improve the public outpatient services.

The online survey will take about 5 to 10 minutes to complete, so please consider carefully and decide whether or not to participate. Click on the next page for a detailed informed consent, so please read it carefully before you start answering.

If you do not wish to participate in this survey, please click on the [quit] to leave this page. Thank you for your help in completing this study.

Sincerely, Dr. Ming-Huwi Lin,

Department of Family Medicine

Taipei Veterans General Hospital

Part I Outpatient Medical Experience

Please select from a list based on your outpatient experience over the last two years	Very Good				Very Bad	No experience
1. Experience in attending primary care clinics	5	4	3	2	1	0
2. Experience in attending general hospitals	5	4	3	2	1	0
3. Experience in attending medical centers	5	4	3	2	1	0

4. Do you have a primary care family doctor that you trust? □1.Yes □2.No
5. When you are sick and don't know which doctor you should see, you will give priority to.
   □1) Primary Outpatient Clinic □2) General Hospital Outpatient Clinic □3) Medical Center Outpatient Clinic
6. When you have acute discomfort symptoms, your first choice would be.
   □1) Primary Outpatient Clinic □2) General Hospital Outpatient Clinic □3) Medical Center Outpatient Clinic
7. The Health Insurance Department has increased the co-payment on you in respect of non-referral to outpatient clinics. □1) Will change outpatient medical practice□2) Will maintain the original outpatient medical practice
8. Do you agree that only minor illnesses should be treated in small clinics and major illnesses should be treated in medical centers?

□1) Yes, for the following reasons (multiple choice)	□A. Reasonable medical consultation can save medical resources effectively.<br>□B. Avoiding overcrowding of seriously ill patients in medical centers for minor illnesses<br>□C. Illness should have started with the family doctor who knows you best.<br>□D. Doctors in medical centers should spend their time caring for inpatients.
□2) Disagree, for the following reasons (multiple choice)	□A. The average patient is incapable of judging the severity of the illness.<br>□ B. Fear of inexperience of primary care doctors and worry of medical conditions<br>□C. Lack of trusted clinics in their community<br>□D. Usually go to a large hospital for medical consultation and treatment.<br>□E. The number of days of prescription in primary clinics is too short

Part II factors considered when selecting an outpatient facility

I . What are your considerations for choosing an outpatient facility when you are experiencing discomfort?	Very important				Very unimportant
1. consider the severity of the disease	5	4	3	2	1
2. consider the visibility of the medical institution (clinic or hospital)	5	4	3	2	1
3. consider the image and reputation of the hospital in the media or social networking sites	5	4	3	2	1
4. whether the medical institution is conveniently located and easily accessible.	5	4	3	2	1
5. Whether the institution has advanced equipment	5	4	3	2	1
6. whether the quality of the drugs in the medical institution is trustworthy	5	4	3	2	1
7. whether the medical institution has diverse specialties	5	4	3	2	1
8. whether the medical institution has a lower co-payment	5	4	3	2	1
9. whether the physician at the facility is willing to write a prescription for a chronic illness.	5	4	3	2	1
10. the friendliness of other medical personnel in the medical institution.	5	4	3	2	1
11. ask your friends and relatives if they recommend you	5	4	3	2	1
12. past medical experience	5	4	3	2	1
13. the waiting time will not be too long	5	4	3	2	1
14. whether the physicians have a good medical practice	5	4	3	2	1
15. whether the doctor's attitude is friendly	5	4	3	2	1
16. whether the physicians are not in a hurry	5	4	3	2	1
17. did the doctor explain the answer in detail?	5	4	3	2	1

18. whether the doctor is trustworthy	5	4	3	2	1
19. visibility of doctors	5	4	3	2	1
20. doctors' image and reputation in the media or social network	5	4	3	2	1

Part III - Basic Information

1. Do you have a chronic disease that requires you to go to the outpatient clinic regularly for medication: □1) Yes □2) No
2. Does a family member who lives with you or knows you well have a chronic illness that requires regular visits to the clinic for medication: □1) Yes □2) No
3. Where do you live?

1) Taipei City
2) New Taipei City
3) Taoyuan City
4) Taichung City, Taiwan
5) Tainan City, Taiwan
6) Kaohsiung City
7) Beyond the Sixth City, Northern Taiwan
8) Beyond the Sixth City, Central Taiwan
9) Southern Taiwan Beyond the Sixth City
10) Yilan Region
11) Other parts of Outlying Islands

4. What is your year of birth?
5. Your level of education.
   □1)Elementary school □2)Junior high school □3) Senior high school □4)College, university □5)Graduate school or above
6. Please fill in your gender: □ 1) Male □ 2) Female
7. What is your current marital status? 1) Unmarried □ 2) Married □ 3) Others
8. What is your current occupation?

1) Medical related
2) Military and Public Education (excluding Medical)
3) Business
4) Industrial and agricultural fisheries
5) Service Industry
6) Free Trade
7) Housekeeping
8) Students
9) Retired
10) Other

9. What is your personal average monthly income?
   □ 1) Less than 15,000 □ 2) 15,001~30,000 □ 3) 30,001~50,000
   □ 4) 50,001~70,000 □ 5) 70,001~90,000 □ 6) Over 90,001

That's the end of the questionnaire, thank you so much for your patience!

If you have questions about Taiwan's medical care and referral system, please contact us. Your valuable suggestions are welcome.
