# Supplementary material for: Why people select the outpatient clinic of medical centers: a nationwide analysis in Taiwan
Source: PeerJ. 2020 Aug 27;8:e9829. doi: 10.7717/peerj.9829 (PMC7456533; doi:10.7717/peerj.9829)
Supplement: Supplemental Information 5 [file peerj-08-9829-s005.docx]

**Supplemental Table 1: Checklist for Reporting Results of Internet E-Surveys (CHERRIES)**

| **Item Category** | **Checklist Item** | **Explanation** |
| --- | --- | --- |
| **Design** | Describe  survey design | An online anonymous survey utilizing self-constructed questionnaire. The main purpose of this study was to evaluate the factors contributing to the patients’ selection of the outpatient clinic of medical centers without a referral. By using the snowball sampling method, the questionnaire was introduced to a variety of community groups. To maximize public outreach, the survey was promoted in different social media with interested citizens being invited to complete the questionnaire and the respondents who took the survey being asked to continue inviting their friends to participate in the survey and fill out the questionnaire. |
| **IRB approval and informed**  **consent process** | IRB approval | This study was approved by the Institutional Review Board of Taipei Veterans General Hospital (2017-07-009AC) |
|  | Informed consent | Informed consent was requested from all participants on the first page of the questionnaire. Only participants who were at least 20 years old and were able to read Chinese fluently were given access. |
|  | Data protection | No personal identifying information was collected. All data are stored on a secure server. Only the researcher can access these materials. |
| **Development**  **and**  **pre-testing** | Development  and testing | Based on a literature review and the focus group, factors that related to the outpatient choice were proposed and included in the questionnaire. The main dependent variable of this study was "preferred choice of outpatient clinics when you are ill," and the independent variables were assessed using the following question: "Please indicate the importance of each of the following factors in your selection of an outpatient clinic when you were ill?” |

| **Development**  **and**  **pre-testing** | Development  and testing | The survey questions were formatted as short answer, single choice, or 5-point Likert rating scale questions.  At the end of the questionnaire, respondents were asked to provide demographic information and information on past experiences during outpatient visits at different hospital levels, attitudes towards copayment, and whether they have a regular family physician. Five experts with expertise in subject content were invited to modify the questionnaire for ensuring content validity. Questions were refined after feedback and finalized into the online survey.  At the beginning of the study, the questionnaire was pretested in 20 patients to determine if the content was appropriate and to ascertain whether the content was understandable. The internal consistency reliability test was used for reliability analysis. Cronbach’s alpha of the questionnaire was 0.895, which is satisfactory. |
| --- | --- | --- |
| **Recruitment process and**  **description of the sample**  **having access to the**  **questionnaire** | Open survey versus  closed survey | The survey was open to participants who was older than 20 years. Participants recruited through the online Google form were not required to register or log in. |
|  | Contact mode | the survey was promoted in different social media with interested citizens being invited to complete the questionnaire. No contact was made with participants outside of the Internet-based survey. |
|  | Advertising the survey | There were no Advertisment for the survey.  The respondents who took the survey being asked to continue inviting their friends to participate in the survey. |
| **Survey**  **administration** | Web/E-mail | The questionnaire was developed in Google form. The hyperlink of the questionnaire was embedded in out website. |

| **Survey**  **administration** | Context | The questionnaire was introduced to a variety of community groups. In all counties in Taiwan, similar open community platforms have been established, and members can post the latest information after simple registration.  To maximize public outreach, the survey was promoted in different social media such as Facebook, Line and the most popular bulletin board system with interested citizens being invited to complete the questionnaire. |
| --- | --- | --- |
|  | Mandatory/vol  untary | The survey was completely voluntary. Users could  access the websites without completing the survey. |
|  | Incentives | No rewards were provided to participants. |
|  | Time/Date | The survey was available online from September to October 2018. |
|  | Randomization of items or  questionnaires | Survey items were not randomized as each subsequent section of the survey intentionally built  on the prior section. |
|  | Adaptive  questioning | Adaptive questioning was programmed into the survey to reduce the complexity of the questions. |
|  | Number of  Items | The final survey tool contained 38 total possible questions and took an average of 5—10 minutes to complete. An average page contained 6-10 questions (range 1-10 questions per page depending on question length) |
|  | Number of screens  (pages) | The total number of pages a participant could see was 5. |
|  | Completeness check | All questions items provided a non-response option  (either “not applicable” or “decline to answer”) Each question required a response in order to advance to  subsequent question screens. A completeness  check was performed after the questionnaire was submitted. |
|  | Review step | A “back” button was provided if participants wished to edit previous answers. |
| **Response**  **rates** | Unique site  visitor | Google analytics was used to calculate unique site  visitors with a cookie-based tracking system |

| View rate  (Ratio unique site visitors/unique survey visitors) | | Not provided.  Requires counting unique site visitors to the first page of the survey, divided by the number of unique site visitors |
| --- | --- | --- |
| Participation rate  (Ratio unique survey page visitors/  agreed to participate) | | For the internet survey, 5060 people browsed the online survey, and 1003 responded and completed the online questionnaire. The response rate was 19.8% |
| Completion rate (Ratio agreed to participate/finished survey) | | 987 out of these 1003 (98.4%) finished the survey. |
| **Preventing**  **multiple entries from the same individual** | Cookies used | Google analytics was used to calculate unique site  visitors with a cookie-based tracking system |
|  | IP check | IP addresses were not collected from participants because of technical difficulties. |
|  | Log file  analysis | No usernames were required to complete  the survey, however, no participant incentive was provided, decreasing the likelihood of completing the  survey multiple times. |
| **Analysis** | Handling of  incomplete questionnaires | Questionnaires with at least 90% of applicable questions completed were analyzed.  Sociodemographic questions were inserted at the end of the survey. |
|  | Questionnaires  submitted with an atypical  timestamp | Time stamps were collected at start of the survey.  The length of time required to complete the survey were not be recorded. It is difficult to identify short completion rates (under 3 minutes –would suggest random “click-through”. |
|  | Statistical  correction | No statistical correction procedures or weightings  were used in the analysis. |

Eysenbach, G. (2004). Improving the quality of web surveys: the checklist for reporting

results of internet e-­‐surveys (cherries). *Journal of medical Internet research*, 6(3)e34

doi:10.2196/jmir.6.3.e34.<http://www.jmir.org/2004/3/e34/>
